# Spatiotemporal dynamics of soil loss and sediment export in Upper Bilate River Catchment (UBRC), Central Rift Valley of Ethiopia

**DOI:** 10.1016/j.heliyon.2022.e11220

**Published:** 2022-10-25

**Authors:** Chakoro Tamire, Eyasu Elias, Mekuria Argaw

**Affiliations:** a Center for Environmental Science, College of Natural and Computational Science, Addis Ababa University, PO Box 1176, Addis Ababa, Ethiopia; b Department of Environmental Science, College of Agriculture, Wachemo University, PO Box 667, Hossana, Ethiopia

**Keywords:** Central rift valley, InVEST SDR model, Sediment export, Soil loss, Conservation prioritization

## Abstract

Soil loss is one of the major challenges for agricultural production in the Ethiopian highlands. The rate and distribution of soil loss (SL) and sediment export (SE) are essential to map degradation “hotspot” areas for prioritizing soil and water conservation measures. The objective of this study was to estimate the dynamics of SL and SE in the Upper Bilate River Catchment of Central Ethiopia. The Sediment Delivery Ratio (SDR) module of the Integrated Valuation of Ecosystem Services and Trade-offs (InVEST) model was used to estimate and map SL and SE. The primary input data were rainfall, soil data, land use, and other biophysical parameters of the study area. The model output confirmed that the average total soil loss of the catchment was 36.8 million ton/yr. It is modeled that soil loss doubles within 30 years. The average annual sediment export was about 3.62 ton/ha/yr. The mean annual soil loss of the study area was 23 ton/ha/yr, which exceeded the soil loss tolerance (SLT), estimated to range between (2–18 ton/ha/yr) in Ethiopia. Based on the soil erosion risk level, about 22% of the catchment area was classified as severely degraded, while 62 % was moderately degraded. Severe soil erosion prevails in the sub-watershed (SW)-5, SW-4, and SW-13. Therefore, these sub-watersheds need priority conservation action to restore the ecosystem processes of the study area.

## 1 Introduction

Soil loss is a key environmental challenge facing the world (Jahun et al., 2015; Maliqi and Singh, 2019). It is an ecological process of detachment, transportation, and deposition of soil materials by erosive agents, mainly rainfall, wind, and gravity (Aksoy et al., 2019; Gadisa and Midega, 2021). Soil loss and sediment export are direct products of the complex interactions between natural (e.g., rainfall, topography, soil characteristics, etc.) and anthropogenic factors such as agricultural land use, deforestation, and urbanization (Phinzi and Ngetar, 2019; Tesema, 2015).

Generally, soil loss and sediment export negatively affect ecosystem services and functions (Degife et al., 2021; Yohannes et al., 2021). It aggravates rocky desertification (Guo et al., 2022). Soil loss affects agricultural productivity (Wolka et al., 2015). That affects the rural population's food and nutrition security (Degife et al., 2021; Haregeweyn et al., 2017; Yigez et al., 2021). It also causes environmental and socio-economic problems due to sediment load in the downstream area. For example, the deposition in the water body can increase the death of aquatic life and water quality pollution. In addition, increasing the cost of managing the water body, shortening the water reservoir’s life span, and flood occurrence can be other examples of socio-economic problems due to the deposition (Yigez et al., 2021).

Ethiopia is one of the sub-Saharan countries that experience severe soil erosion, with an average annual loss of 16–300 ton/ha/yr in cultivated lands (Hurni, 1988). It results in food insecurity and a loss of 2–3% of agricultural GDP (Kirui and Mirzabaev, 2009; Yesuf and Ringler, 2008). Factors such as Slope steepness, long cultivation history with outdated technology, and overgrazing make soil erosion more severe (Basin et al., 2019).

The Upper Bilate River Catchment (UBRC) is one of the areas that experience severe soil erosion in Ethiopia (Gadisa and Midega, 2021). Sheet or overland soil erosion is the dominant type of erosion in high lands of Ethiopia, including this catchment area (Haregeweyn et al., 2015). A study in Ethiopia's Central Rift Valley (CRV) indicated that annual soil erosion rates increased from 31 ton/ha in 1973 to 56 ton/ha in 2006 (Gadisa and Midega, 2021; Meshesha et al., 2012). The UBRC on the CRV's escarpment contributes to the high erosion rates in the rift valley lakes basin. However, the Spatio-temporal dynamics of soil loss and sediment export are hardly studied in the catchment, hindering the development of site-specific strategic planning for soil and water conservation and sustaining a planned project across the catchment area. In addition, there are limitations in scientific reasoning and prioritizing the conservation area using geospatial analysis approaches (Haregeweyn et al., 2015). Estimating soil loss and sediment export has a vital role in soil and water conservation (Degife et al., 2021; Kumar and Singh, 2021). It helps to plan and forecast its level of impact and allows us to design better structures and policies to reduce the loss rate and effects on downstream irrigation, water treatment, recreation, and reservoir performance (Degife et al., 2021; Sharp et al., 2020).

Estimating soil loss and soil load is challenging due to the complex interdependency between the status of humans and the biophysical parameters. In this regard, researchers have widely used the RUSLE model to estimate soil erosion. However, the model is unable to estimate the amount of sediment export that reaches the water body. On the other hand, the InVEST SDR model has an advantage in addressing the limitation of the RUSLE model (Girma and Gebre, 2020; Jakubínský et al., 2019). The model has the capacity to estimate the rate of soil loss, sediment export, sediment retention, and other erosion process components. The objectives of this study were: (1) to model the spatiotemporal trend of soil loss and sediment export; (2) to estimate and map the annual rate of soil loss and sediment export; (3) to analyze the hotspot area for soil erosion and a priority area for conservation. The outcomes are expected to be helpful in understanding the rate and trends of soil erosion and planning ecological interventions before irreversible damage occurs.

## 2 Materials and methods

### 2.1 Description of the study area

**Figure 1:**
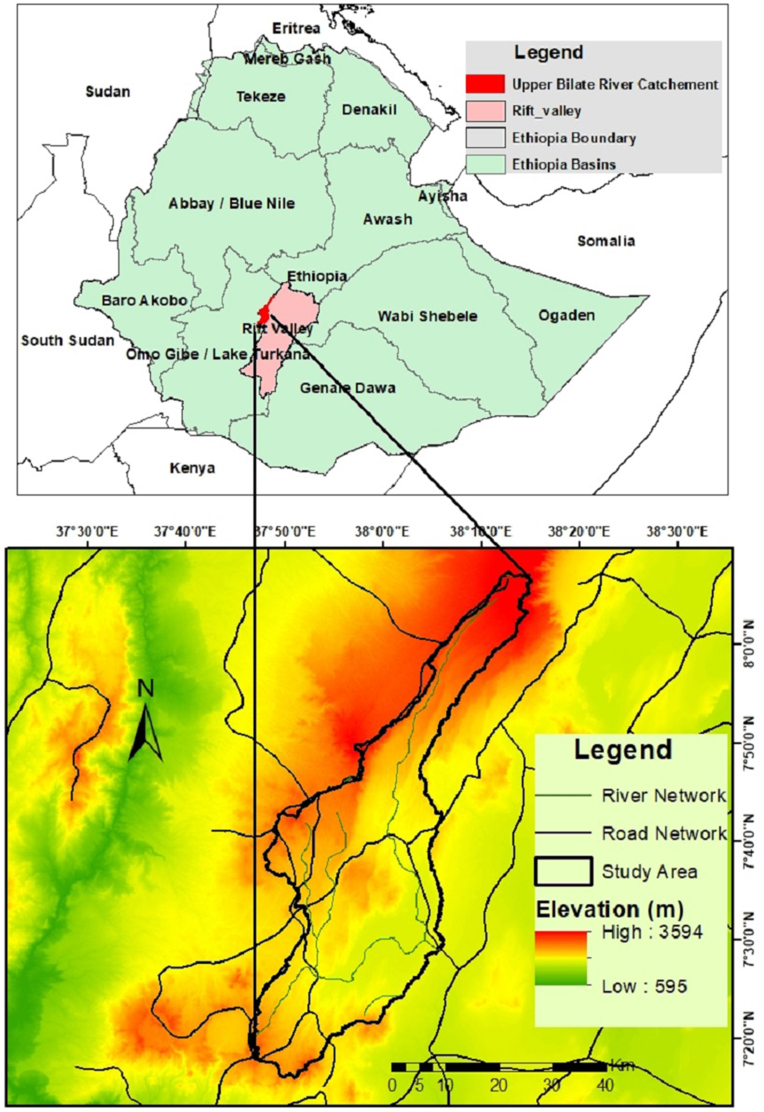
Location map of the study area.

Upper Bilate River Catchment (UBRC) is found in the Western Escarpment of the Main Ethiopian Rift. It can also be called Boyo catchment. Geographically, it is located between 37°30′0″E–38°30′0″E and 7°10′0″N–9°00′0″N encompassing an area of 1670 km^2^ (Figure 1). Administratively, it falls within the administrative zones of Hadiya, Gurage, Silte, Kembata Tembaro, and Alaba Special Woreda of the Southern Nations Nationalities and People Regional State (SNNPR). The elevation ranges from 1800 to 3400 m.a.s.l.

**Figure 2:**
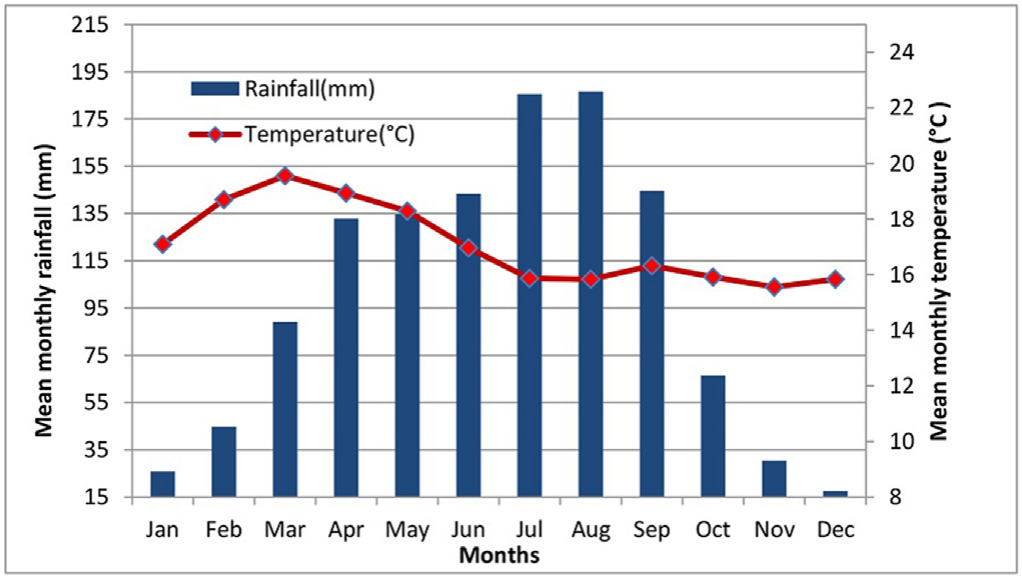
Mean annual temperature and mean annual rainfall in the UBRC (1987–2019).

Based on the observed 30 years of climate data (1989–2019) obtained from the weather stations in the UBRC, including Alaba, Angecha, Butajira, Fonko, Hossana, Imdbir, Wolkite, and Wulbareg of the National Meteorology Agency of Ethiopia (NMA) and plotted in Figure 2 (NMA, 2021). The mean annual rainfall and mean annual temperature of the study area are 1272 mm and 17.6 °C, respectively. The mean maximum and minimum temperatures are 25.62 °C and 11.88 °C, respectively. The monthly mean maximum temperature peaks in March (27.73 °C) and the lowest point in December (10.8 °C).

Based on the collected and analyzed data, the area receives unimodal rainfall distribution in which the rain becomes at its peak in July and August. The dry season extends from October through March in the area. Weira and Guder are two perennial rivers that feed into the Bilate River. Flooding is expected in the catchment area, mainly in Shashogo wereda, Silti wereda, and Alaba woredas during the rainy season. Different soil and water conservation measurements such as stone bunds, terracing, and mulching has been implemented in the area though the success is questionable. The major annual crops are wheat, barley, teff, and maize. The site is also widely known for growing perennial crops, including enset, coffee, and chat. Enset crop is a staple food for the catchment area (Tamire and Argaw, 2015).

### 2.2 Description of InVEST SDR models

The InVEST Sediment Delivery Ratio Model (SDR) model is one of the Natural Capital projects (Sharp et al., 2020). The SDR model is an InVEST empirical model commonly used to estimate soil loss potential by water and sediment deposition that reaches the stream in the catchment (Sharp et al., 2020). This model simulates erosion using the Revised Universal Soil Loss Equation (RUSLE). The InVEST model is more flexible and less data-intensive than other hydrological models like the Soil and Water Assessment Tool (SWAT). It can analyze soil loss and sediment export from each land use type (Hamel et al., 2015). It can also estimate the amount of sediment that reaches the water bodies and others (Aneseyee et al., 2020; Sharp et al., 2020).

### 2.3 Soil loss estimation

Soil loss simulation was done by using the InVEST SDR model based on the Revised Universal Soil Equation/RUSLE model equation that was proposed by Wischmeier and Smith (1978). RUSLE is commonly used to estimate soil loss from watersheds having different or similar land use (Boufala et al., 2020; Girma and Gebre, 2020). It is designed to assess soil loss carried by runoff from specific field slopes in specified cropping and management systems (Panditharathne et al., 2019).

As a function of five independent parameters, RUSLE calculates the soil loss through sheet and rill erosion (Wischmeier and Smith, 1978). The input factors are rainfall erosivity, soil erodibility, the topography of the area (length and gradient), cover management, and conservation practice. The loss rate was simulated for 1991–2021 by focusing on the dynamics of LULC change. The equation is presented as follows in Eq. (1).

(1) $$rusle_{i}={\left(R*K*LS*C*P\right)}_{i}$$

where A = computed average annual soil loss in tons ha^−1^ year^−1^; R = rainfall-runoff erosivity factor (MJ mm/ha/hr/yr); K = soil erodibility factor (ton/ha h/MJ/ha/mm); LS = slope length and steepness factor (dimensionless); C = cover management factor (dimensionless, ranges from zero to one); P = conservation practice factor (dimensionless, ranges from zero to one).

Cell statistics were used to produce the mean annual soil loss of the study area based on 1991, 2001, 2011, and 2021 loss rates.

### 2.4 Sediment export

Annual sediment export is the proportion of soil loss reaching the nearby streams (Sharp et al., 2020). Two major steps had applied for the analysis of sediment export. Initially, the connectivity index (CI) for each pixel was analyzed based on the work of (Borselli et al., 2008). The connectivity index explains the hydrological linkage between sources (from the landscape) and sinks (like streams) of sediment. As written in Eq. (2), IC is a function of Dup-area upslope of each pixel and Ddn-flow path between the pixel and the nearby stream.

(2) $$IC=\log_{10}\left(\frac{Dup}{Ddn}\right)$$

Dup is upslope component and which is given by:

(3) $$Dup=\bar{C}\;\bar{S}\sqrt{A}$$

where, C¯ is the an average C factor of the upslope contributing area; $\bar{S}$ is the average slope gradient of the upslope contributing area (m/m); and A is the upslope contributing area (m^2^). The upslope contributing area was delineated from a Multiple-Flow Direction algorithm. The downslope component (Ddn) is defined as:

(4) $$Ddn=\sum_{i}\frac{di}{CiSi}$$

where, di is the length (m) of the flow path along the ith cell based on the steepest downslope direction; Ci and Si represent the C factor and the slope gradient of the ith cell, respectively.

The second step was to compute the SDR ratio for a pixel i from the connectivity index (IC) based on Vigiak et al. (2012) as written in Eq. (5).

(5) $$SDRi=\frac{SDR_{\max}}{1+\exp\left(\frac{ICo-ICi}{k}\right)}$$

where, SDR_max_ is the maximum theoretical SDR set to an average value of 0.8 (Vikiak et al., 2012), and IC0 and k are calibration values that determine the shape of the SDR-IC relationship (increasing function) (Sharp et al. 2020).

The sediment export from given pixel i, Ei (ton/ha/yr) is given by:

(6) $$Ei=usle_{i}*SDR_{i}$$

The total sediment export from the watershed, E (ton/ha/yr), is given by:

(7) $$Ei=\sum_{i}Ei$$

### 2.5 Analysis of input data for InVEST SDR model

The main data source for the InVEST SDR model to estimate soil loss and sediment export was satellite imagery, digital elevation model (DEM), soil data, meteorological data, and hydrological data. Based on this, R-factor, K-factor, biophysical table (C-factor and P-factor), Land use data, DEM data, and catchment boundary were used as input for the model.

#### 2.5.1 Land Use data

Four Landsat imageries with 30 m resolutions were used for the SDR model’s LULC change analysis and inputs. The imagery for the study years (1991, 2001, 2011, and 2021) was downloaded from the website of the United States Geological Survey (USGS) (https://earthexplorer.usgs.gov/). The detailed information for each Landsat is presented in Table 1. The data were downloaded in the dry season with less than 10% cloud cover. Pre-processing of classification, such as image correction, projection, layer stacking, and mosaicking, was done using the software QGIS 3.16.11 (Sharp et al., 2020).

**Table 1:** Information on Landsat data.

Year	Satellite	Sensors	Resolution(m)	Path/row	Date of acquisition
1991	Landsat 5	Thematic Mapper TM	30	169/55 &54	1991-12-12
2001	Landsat 5	Thematic Mapper TM	30	169/55 &54	2001-11-05
2011	Landsat 5	Thematic Mapper TM	30	169/55 &54	2011-01-14
2021	Landsat 8	Operational Land Images OLI	30	169/55 &54	2021-01-28

Supervised classification was undertaken for this study using the Maximum Likelihood Algorithm. It is one of the commonly used algorithms in QGIS. It assumes a normal distribution of cells in each class. By this assumption it helps to determine the class into which it most likely belongs. It was carried out by collecting 385 sample sites as a region of interest (ROI) from all the classes. On average, 55 signature sample was collected, that is in the acceptable range (10–100) (Schowengerdt, 2007). The study area was classified into seven (7) LULC classes as presented in Table 2 (water body, vegetation area, cropland, Enset homestead, grazing land, bare land, and built-up area).

**Table 2:** LULC types and their description.

LULC	Description
Water Body	The area includes Lakes, Rivers, and ponds (natural and artificial)
Vegetation Area	The area covers forest land, shrub land, and grassland
Crop Land	Cultivated land mainly annual cropland area, Cereal crop area
Enset-homestead	It mainly includes the Enset area, banana, coffee, and other agroforestry
Grazing Land	The area includes mainly open space, grazing land, and wetlands
Settlement	It includes urban and rural built-up areas, paved roads, and other infrastructure (transport and industrial facilities)
Bare Land	The area includes rocky areas, bare soil, and eroded land

To assesses the accuracy of the classification, statistical methods like overall accuracy and kappa value were applied. Ground control point and google earth were used as base data for accuracy assessment for the recent year (2021) and the other years, respectively. The overall accuracy and Kappa value were above 85% and 0.8 for all the years, respectively.

#### 2.5.2 Rainfall data

The Rainfall erosivity (R) factor is one of the input data for the SDR model in the InVEST software which is derived from mean annual rainfall. The R factor is the power of rain to initiate soil erosion (Jahun et al., 2015). In fact it is the potential ability of rain to cause soil erosion (Jahun et al., 2015). It represents the erosive force of specific rainfall events in a given area or represents the numeric power of the rainfall (Wischmeier and Smith, 1978). Therefore, rainfall's amount, intensity, and distribution can determine erosivity (Tesema, 2015; Wolka et al., 2015).

Monthly total rainfall from eight weather stations was collected from the Ethiopian Meteorological Agency and analyzed for mean annual rainfall (NMA, 2021). Different empirical equations for rainfall erosivity have been developed. Based on the data availability and climatic condition the equation may vary from area to area. Some use daily data; others use annual data based on their availability of data. In this work, the R-Factor was calculated in the ArcGIS raster calculator by using (Eq. (8)), established by Hurni (1985) for Ethiopia (Gashaw et al., 2018; Wolka et al., 2015). Inverse Distance weighted (IDW) in ArcGIS spatial analysis was used to generate erosivity value across the study area.

(8) R=−8.12+(0.562*P)

where, *R* is the rainfall erosivity factor; *P* is the mean annual rainfall (mm).

#### 2.5.3 Soil data

The soil Erodibility (K) factor is one of the main factors that govern soil erosion (Girma and Gebre, 2020). It measures the susceptibility of soil particles through detached and transported by rainfall and runoff (Wawer et al., 2005). It is highly dependent on the properties of the soil (texture, structure, organic matter contents) (Wischmeier and Smith, 1978). Therefore, it expresses its inherent resistance to particle detachment and transport by rainfall.

Thus, the soil color-type approach was used for this study, as Hurni suggested by Hurni (1985). The soil unit map was extracted from the Rift Valley Basin Authority database The main soil types are Pellic Vertisols (8.2%), Eutric Nitosols (10.3%), Lithosols (14.1%), Chromic Luvisols (48.3%), Molic Adndosols (13.5%) and Dystric Nitosols (5.5%). The soil colors and their corresponding K-value were obtained from other published literature (Bekele and Gemi, 2021; Gashaw et al., 2021; Hurni, 1985).

#### 2.5.4 Digital elevation model (DEM)

The LS – factor is a topographic factor, a combination of a factors of slope length (L) and slope steepness (S) (Wischmeier and Smith, 1978). Computation of the LS – factor from a DEM with 30 m spatial resolution was used as input InVEST SDR model. The combined effect of the slope length and gradient determine the volume and the rate of soil erosion (Degife et al., 2021; Gashaw et al., 2018; Wischmeier and Smith, 1978). The higher value of the LS factor of the catchment, the higher will be rate power of soil erosion (Jahun et al., 2015; Wischmeier and Smith, 1978). LS factor calculation that was developed by Wischmeier and Smith in the original USLE method was limited to a small area in uniform slope and gradients (Wischmeier and Smith, 1978). Thus, LS – factor calculation used for this research was Desmet and Gover's techniques (Eq. (9)) (Desmet and Govers, 1996).

(9) $$LSi=Si\frac{{\left(Ai-in+D²\right)}^{m+1}-Ai-{in}^{m+1}}{D^{m+2}*xi^{m}*22.13^{m}}$$

where, Si represents the slope of a grid cell computed as a function of slope radians θ, with S = 10.8 * sin(θ) + 0.03 for θ<9% while S = 16.8 * sin(θ)–0.50 for θ ≥ 9%; Ai−in represents the contributing area in (m^2^) at the inlet of a grid cell which is computed based on the multiple flow direction method; D indicates the grid cell linear dimension in m; xi is the mean of aspect weighted by the proportional outflow from grid cell i determined by a Multiple-Flow Direction algorithm. It is calculated by ∑d∈ {0, 7}*Pi (d)/xd, where xd = |sinα(d)| + |cosα(d)|; where α(d) is the radian angle for direction d and Pi(d) is the proportion of total outflow at cell i in direction d; m is the length exponent of the LS factor, that is based on the classical USLE, as discussed in (Olivera et al., 2013), where: m = 0.2 for slope ≤ 1%, m = 0.3 for 1% < slope ≤ 3.5%, m = 0.4 for 3.5% < slope ≤ 5%, m = 0.5 for 5% < slope ≤ 9%, and m = β/(1 + β) where β = sin θ/0.0986/(3 sin θ0.8 + 0.56) for slope ≤ 9%.

#### 2.5.5 Biophysical table

Biophysical factors include the data of cover management (C-factor) and support practices (P factor) corresponding to each land use land cover class (LULC) by assigning the same Land use code/LUCODE for each land-use class. The C-factor is the most important factor in the RUSLE model due to its representation in reducing soil erosion, as stated by (McCool et al., 1995). The cover value (C-factor) for each LULC was collected from different literature suggested for Ethiopia. The C value ranged from 0 to 1, representing water bodies and bare land, respectively.

The P-factors reveal the role of land management and conservation practice in minimizing soil erosion. In this study, P-factor for conservation was collected from published literature. Based on the Wischmeier and Smith (1978) techniques, the spatial land use map of study area was changed in to polygon in Arc Map. Then the polygon was classified into cropland and non-cropland use area (Gashaw et al., 2021). The cropland classes were further categorized into six slope classes because land management activities are highly dependent on slope classes. Then the croplands under each slope ranges were given p-values (0.1, 0.12, 0.14, 0.19, 0.25, 0.35, and 1 for slope range of 0–5, 5–15, 15–20, 20–30, 30–50, 50–100, and >100, respectively) while the remaining non-cropland use were assigned with a uniform default value of 1. The obtained values range from 0 to 1, with lower values denoting somewhat more effective soil erosion control techniques.

**The Watershed boundary** was another input data for the SDR model. Thus, the boundary of the catchment was delineated and extracted from DEM using the Arc SWAT software.

### 2.6 Model validation

The InVEST-SDR model was validated by analyzing the annual observed and simulated data of the sediment export. The observed data were collected from four hydrological stations (Guder, Gombora, Batena and Bilate-Alaba) (MoWE, 2022). For this study, R^2^, PBIAS, RSR, and NSE were used to evaluate the model's performance.

Based on Moriasi et al. (2015), suggestion if the absolute value for PBIAS, RSR, NSE, and R2 is ≤ ±25, ≤ 0.7, ≥0.6, >0.6, then the model has acceptable performance with different levels of performance (Huang et al., 2020; Moriasi et al., 2015). However, when the absolute value for PBIAS, RSR, NSE, and R2 is ≤± 10, ≤ 0.5, ≥0.75, and ≥0.75, then the model's performance is very good. Similarly, when the absolute value for PBIAS, RSR, NSE, and R2 is between ±10 and ±15, between 0.5 and 0.6, between 0.6 and 0.75, and 0.5 and 0.75, then the performance of the model will have good performance (Carlos Mendoza et al., 2021; Moriasi et al., 2015).

## 3 Results

### 3.1 Validation of the model

**Figure 3:**
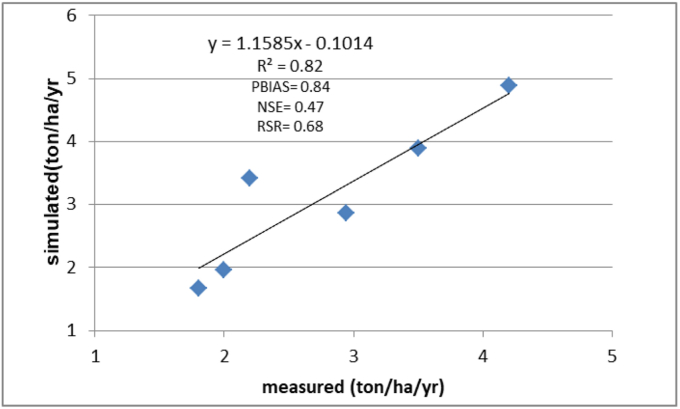
Validation of InVEST model.

The InVEST SDR model was validated by comparing the observed and simulated data of the sediment load. The analysis shows 0.84, 0.81, 0.47, and 0.68 for PBIAS, R^2^, NSE, and RSR, respectively (Figure 3). It can be categorized as very good (PBIAS and RSR) and satisfactory performance (NSE and RSR) (Moriasi et al., 2015).

### 3.2 Spatiotemporal changes of soil loss

**Figure 4:**
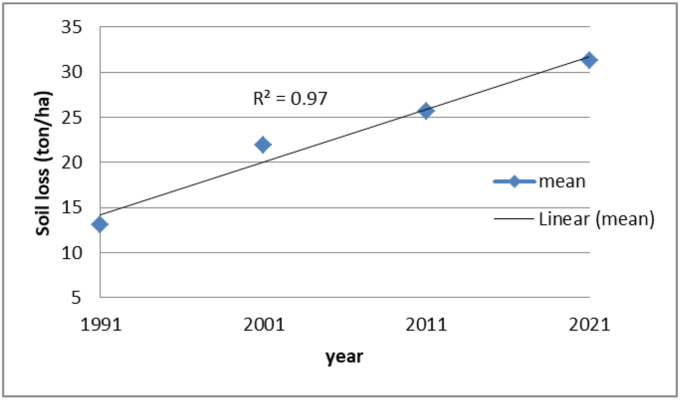
Temporal distribution of the soil loss in the study area.

In order to model the dynamics of annual soil loss rates for the UBRC InVEST SDR model was used by integrated with other models and software (QGIS, ArcMap) (Aneseyee et al., 2020). The total soil loss was changed from 22.06 million ton in 1991 to 44.8 million ton in 2021. This shows us the total loss of the catchment is doubling the amount of loss within 30 years. The mean soil loss was 13.06 ton/ha/yr, 21.89 ton/ha/yr, 25.66 ton/ha/yr, and 31.26 ton/ha/yr in 1991, 2001, 2011 and 2021, respectively. The annual soil loss dynamic is linearly increasing yearly in the study area (Figure 4).

**Figure 5:**
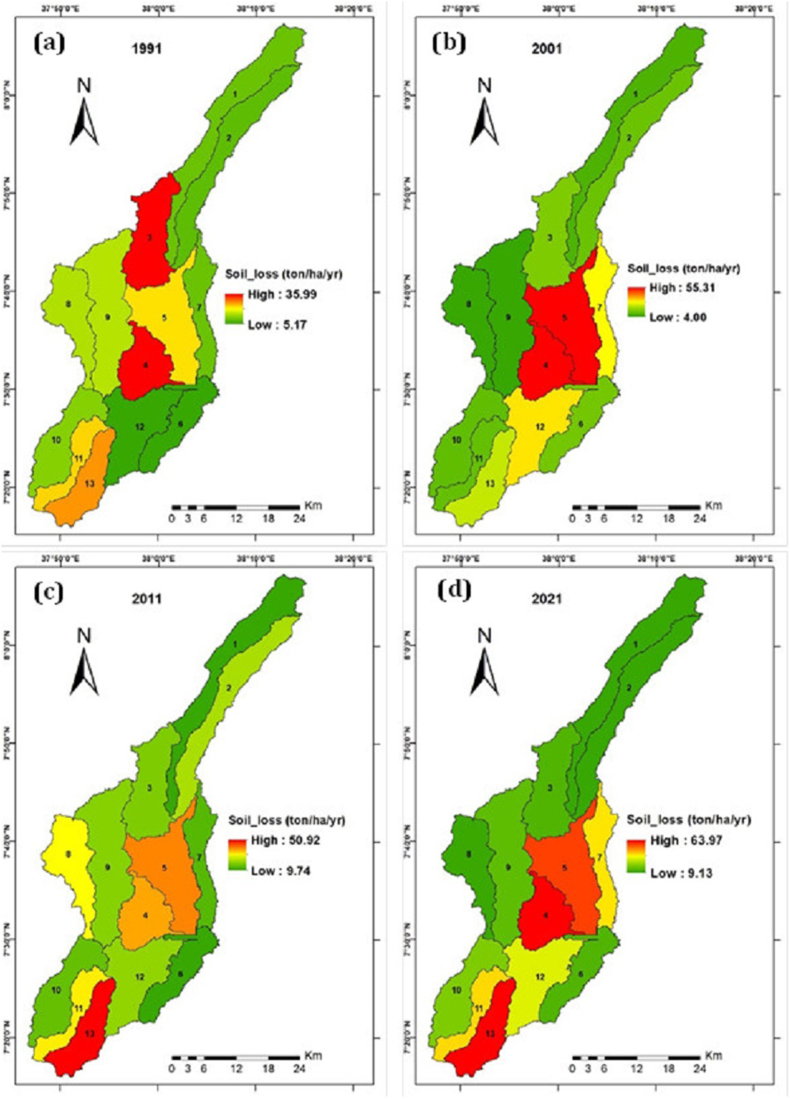
Spatial and temporal distribution of soil loss (1991–2021) (a) 1991; (b) 2001; (c) 2011; and (d) 2021.

Figure 5 presents the spatial distribution of the soil loss in the study area. It indicates the amount of soil loss in ton per hectare in relation to the sub-watershed. From this, we understand that soil loss was significantly increasing in the past 30 years.

The mean annual soil loss of the study area was 23 ton/ha/yr, and the average total soil loss of the catchment was 36.8 million ton/year. The maximum mean annual soil loss was 42.84/ton/ha/yr in SW-5. The minimum mean annual soil loss was 9.44 ton/ha/yr in SW-1.

### 3.3 Hotspots of soil erosion

**Figure 6:**
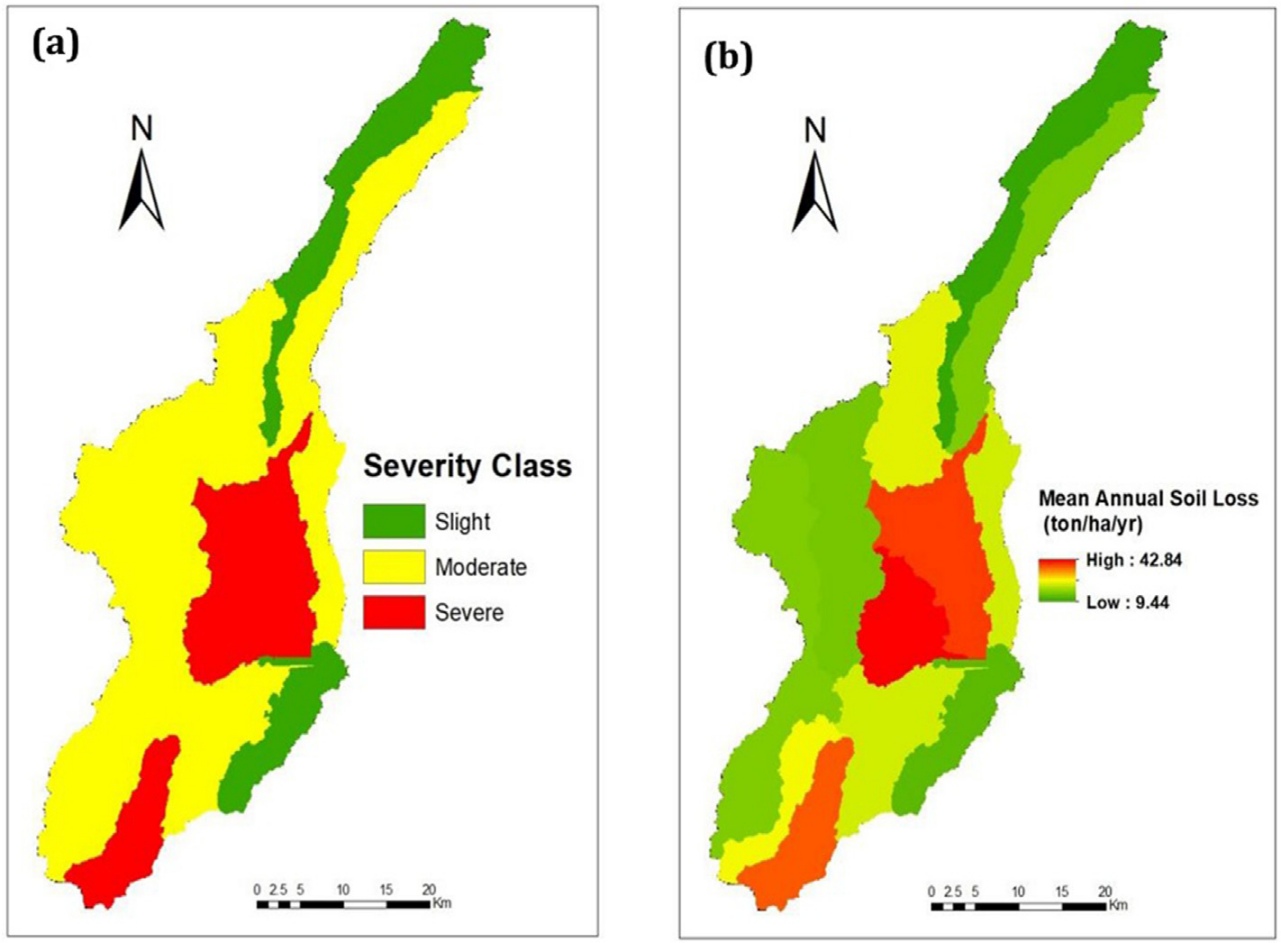
(a) Severity class and (b) Mean annual soil loss rate.

The prioritizing the soil erosion area can be done using the analysing the water holding capacity of soil, compound factor (morohometric, soil character, Geology and land cover), and soil loss rate in Rusle (Kulimushi et al., 2021; Kumar and Singh, 2021; Maliqi and Singh, 2019). The model identified the soil loss hotspot area for immediate intervention and conservation. The severity group and conservation priority were conducted using FAO standards (Haregeweyn et al., 2017). It was based on the soil loss rate in RUSLE (Kumar and Singh, 2021). Based on this, SW-5, SW-4, and SW-13 are grouped in the severe classes or hotspot areas for soil erosion, the red color in Figure 6. From the total area of the catchment, about 22% was included in this class (severe). About 15.08 million tons of soil eroded yearly from this hotspot area (Table 3); it covered 38.6 % of the total soil loss per year.

**Table 3:** Soil loss severity classes and conservation priority.

No	soil loss rates (t/ha/yr)	Severity Class	Area (skm)	%_Area	Average annual loss-	% mean loss	Priority class	SW
2	5–15	Slight	269.7	16.22	10.80	15.50	III	Others
3	15–30	Moderate	1030.6	61.98	19.45	27.92	II	1, 6
4	30–50	Severe	362.5	21.80	39.41	56.58	I	5, 4, 13

As presented in Table 5, the SW-4, SW-5, and SW-13 are needed immediate conservation measures rather than the other sub-watersheds. The average annual soil loss rates and severity classes are arranged and presented in Table 3 and Figure 6.

### 3.4 Sediment export

The SDR model was used to estimate the amount of sediment delivered or sediment exported to the stream in the study area (Aneseyee et al., 2020; Degife et al., 2021; Gashaw et al., 2021; Yohannes et al., 2021). It indicates the rate of sediment that is transported and reaches the river. It can be considered a source of pollution to the water body. The sediment export and soil loss rate in the study years are presented in Table 5.

**Figure 7:**
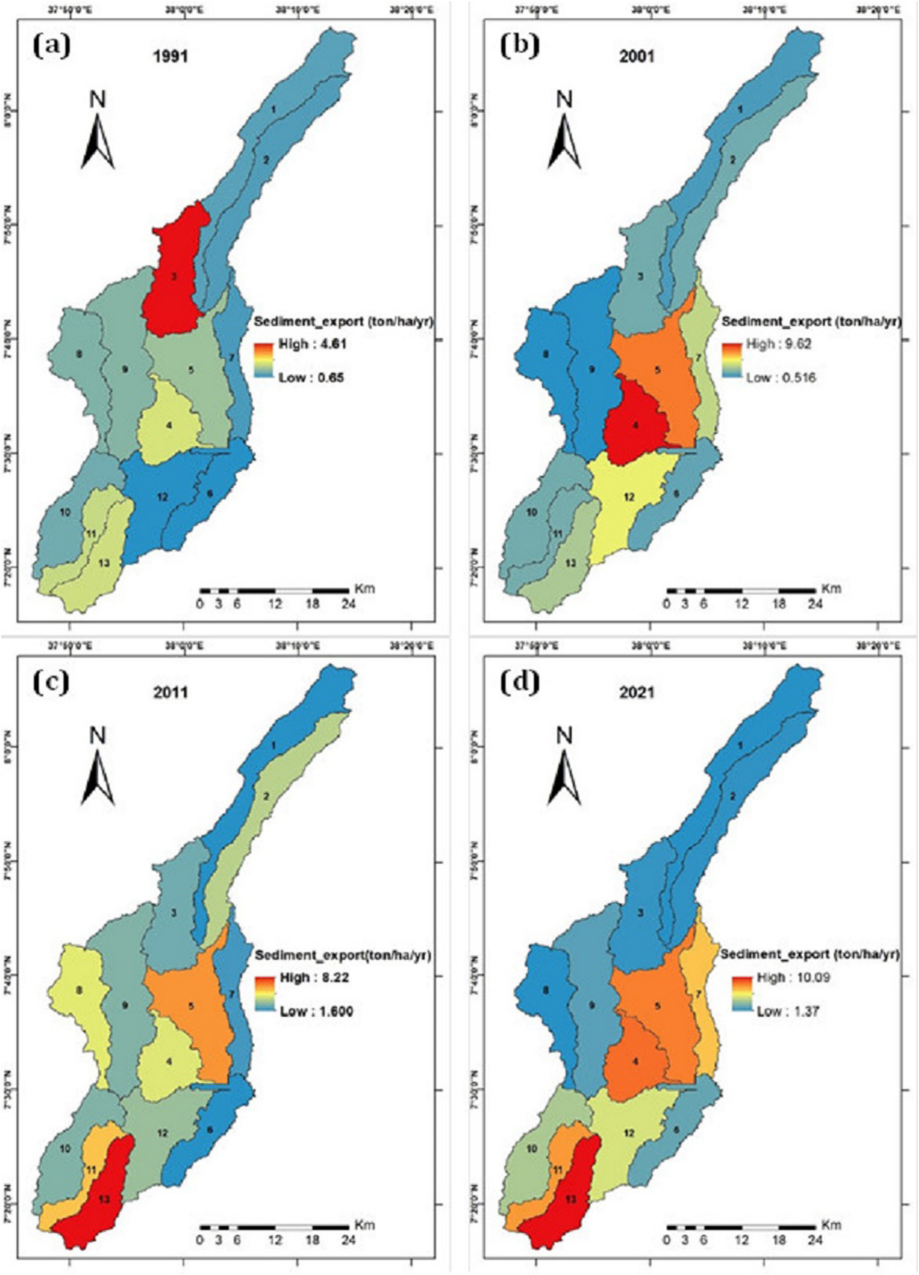
Spatial distribution of sediment export (1991–2021) (a) 1991; (b) 2001; (c) 2011; and (d) 2021.

Similar to the mean annual soil loss rate, mean annual sediment export showed an increasing trend in the last 30 years. It was 1.8 ton/ha/yr, 3.7 ton/ha/yr, 4 ton/ha/yr, and 5 ton/ha/yr in the 1991, 2001, 2011 and 2021, respectively (Figure 7).

In the same way, the total load in each sub-watershed showed an increasing trend in the study period. The average annual sediment export is around 3.62 ton/ha/yr, but the average total sediment export was 5.8 million ton/yr from the study area.

The higher sediment export was found in the SW- 4, SW-5 andSW- 13 with the value of 5.37 ton/yr, 5.74 ton/yr, and 6.11 ton/yr, respectively. Both sediment export and annual soil loss have shared the same sub-watershed with higher rates. That is because the sediment export is the multiplication of the rate of annual soil loss and sediment delivery ratio of the study area.

## 4 Discussion

### 4.1 Spatiotemporal changes of soil loss and sediment export

Considering the rate of soil loss and sediment export in time and place is important during applying scientific-based SWC. As presented in Table 5, the mean annual soil loss was 13.06 ton/ha/yr, 21.89 ton/ha/yr, 25.66 ton/ha/yr, and 31.26 ton/ha/yr in 1991, 2001, 2011 and 2021, respectively. Similarly, the mean annual sediment export was 1.8 ton/ha/yr, 3.7 ton/ha/yr, 4 ton/ha/yr, and 5 ton/ha/yr in 1991, 2001, 2011, and 2021, respectively. These outputs indicate the increment of soil loss and sediment export year to year for the last 30 years. This result agrees with other studies findings on CRV (Gadisa and Midega, 2021; Meshesha et al., 2012). For instance, it was a noticeable increase with annual soil loss rates of 31 ton/ha in 1973 and 56 ton/ha in 2006 in CRV (Gadisa and Midega, 2021; Meshesha et al., 2012). In 1991, 22.06 million ton of total soil was loosed from the catchment, which doubled in the year 2021, with 44.8 million ton of total soil from the same catchment.

The change may be because of land-use change, soil characteristics, and topographic change (Aneseyee et al., 2020; Meshesha et al., 2012). These changes have a consequence in the water body (siltation) and in agricultural activity (washing the upper fertile soil) (Kumar and Singh, 2021; Mulat et al., 2020).

Cropland and bare land showed the highest contribution to the increment of soil loss rate. Both bare land and annual cropland showed an average change of 3.66 % and 9.55 %, respectively, in the study years. This finding is comparable with the result (Aneseyee et al., 2020; Bai et al., 2019; Jakubínský et al., 2019). Similarly, changing the natural vegetation and agroforestry-based agriculture to intensive farming may be another reason for the change in soil loss (Tesema, 2015). Enset is resistant to soil loss perianal crop (Nurebo, 2017; Tamire and Argaw, 2015). However, the enset-related land cover has been replaced with the annual crops.

In addition to the land-use change, the characteristics of the soil have a strong influence on soil erosion. This is similar results to the output (Amsalu and Mengaw, 2016; Aneseyee et al., 2020; Degife et al., 2021; Yohannes et al., 2021). From the analysis, the annual soil loss rate from the Chromic Luvisols and Drystic Nitosols was 29.3ton/ha/yr and 32.8ton/ha/yr, respectively. These soil types have low organic carbon content relative to other soil types, which may be the reason for the more significant contribution of soil loss. On the other hand, the black-colored, Pellic Vertisols soils have a low contribution to the soil loss rate (6.14 ton/ha/yr). In which we can find better organic carbon content. However, this finding is different from the output of Bekele and Gemi (2021) but similar to (Amsalu and Mengaw, 2016).

The maximum and the minimum mean soil loss was 42.84 ton/ha/yr in SW-5 and 9.45 ton/ha/yr in SW-1, respectively, but the mean annual soil loss rate of the catchment was 23 ton/ha/yr. This showed that the area has a higher rate of soil loss, even more than the formation rate (2–22 ton/ha/yr) presented by Hurni (1983) for Ethiopia. A study in China shows the loss rate above 10 ton/ha/yr will not be reversed even within 50–100 years, as cited (Yesuph and Dagnew, 2019). Accordingly, except for the SW-1, the others probably will not be reversed with modest measures. Additionally, the calculated mean annual soil loss rate (23 ton/ha/yr) from the catchment area was above the soil loss tolerable (SLT) (2–18 t/ha/yr) that was stated for Ethiopia by Hurni (1986). This finding is higher as compared to the studies conducted in different parts of Ethiopia by Degife et al. (2021) in the Hawassa watershed (37 ton/ha/yr) (Yesuph and Dagnew, 2019) in Beshillo Catchment (37 ton/ha/yr), and (Bewket and Teferi, 2009) in Chemoga watershed (93 ton/ha/yr). But lower than the findings of Bekele and Gemi (2021) in the Dijo watershed (2.2 ton/ha/yr), Brhane and Mekonen (2009) in the Medego watershed (9.63 ton/ha/yr), and Ayalew (2015) in Zingin watershed (5–11 t/ha/yr). The output is comparable with the result that is reported by Girmay et al. (2020) in the Agewmariam watershed (25 ton/ha/yr) and Haregeweyn et al. (2017) in the Upper Blue Nile River (27.5 ton/ha/yr).

### 4.2 Hotspots and priority sub-watersheds for SWC

The study analysis showed that SW- 5, SW- 4, and SW- 13 are in the severe classes (30–50 ton/ha/yr) (Table 4). The hotspot area comprises about 22 % of the catchment, whereas the moderate severity level includes about 62 % of the catchment. That was based on the FAO severity group standard (Yesuph and Dagnew, 2019). About 15.08 million ton of soil has loosed each year from severe or hotspot areas of the catchment. Accordingly, the severe class (SW- 5, SW-4, and SW- 13) are the first (I) priority areas for conservation, and SW- 1 and SW-6 will follow as the second (II) priority. The SW- 5, SW- 4, and SW- 13 needed the immediate conservation measure. Therefore, undertaking the conservation practice based on the given priority is essential for the sustainability of conservation in the study area. This result and idea are similar to research output by Gashaw et al. (2018) and Tessema et al. (2020).

**Table 4:** The rate of soil loss and sediment export at the sub-watershed level.

SW_ID	Area_skm	Elev	Soil loss		Sediment export		Severity class
			Mean	Total	Mean	Total	
SW-1	174.46	2921.90	9.45	1723297	1.50	273477.10	Slight
SW-2	157.79	2700.83	16.55	2645800	2.46	392522.00	Moderate
SW-3	140.00	2498.88	24.11	3565035	3.22	476462.00	Moderate
SW-4	98.75	2184.26	42.84	4487573	5.38	562936.80	Severe
SW-5	168.33	2162.90	38.79	6930632	5.74	1026235.00	Severe
SW-6	95.27	1960.58	13.31	1296456	2.09	203667.40	Slight
SW-7	86.18	2101.45	22.76	1997401	3.52	309075.60	Moderate
SW-8	118.97	2524.67	16.54	2057684	2.45	304975.80	Moderate
SW-9	197.94	2498.36	15.82	3313513	2.27	475866.20	Moderate
SW-10	117.83	2207.77	16.78	2076271	2.84	351646.60	Moderate
SW-11	64.74	2330.09	25.24	1723095	4.64	317044.00	Moderate
SW-12	147.10	2071.55	22.94	3562375	3.43	533505.90	Moderate
SW-13	95.51	2370.88	36.91	3664298	6.12	607451.30	Severe

**Table 5:** Sediment export and soil loss in 30 years.

Sediment export			Soil loss	
Year	Mean (ton/ha/yr	Total (ton/yr)	Mean (ton/ha/yr)	Total (ton/yr)
1991	1.8	2817970	13.06661	22060491
2001	3.7	5926762	21.89521	36917759
2011	4	6573154	25.66538	43286822
2021	5	6996230	31.26184	44806659

### 4.3 Sediment export

The study reported that the average annual sediment export and an average total sediment export were 3.62 ton/ha/yr and 5.8 million ton/yr in the study catchment. As shown in Table 4, the highest sediment export was found in the sub-watersheds 4, 5, and 13, with the value of 5.37 ton/yr, 5.74 ton/yr, and 6.11 ton/yr, respectively. The sediment export rates can be grouped into five classes; such as very low (0–5 t ha^−1^ yr^−1^), low (5–11 t ha^−1^ yr^−1^), moderate (11–18 t ha^−1^ yr^−1^), high (18–25 t ha^−1^ yr^−1^) and very high (>25 t ha^−1^ yr^−1^) (Gashaw et al., 2021). Based on these classes, the mean sediment export from the entire catchment was grouped in the very low range. The SW- 4, SW-5, and SW-13 were also in the low range. However, the researchers have observed the sediment accumulation on the riverside from the agricultural area-during field visit.

The result is comparable with the result of (Gashaw et al., 2021) and greater than the result of (Degife et al., 2021). Like soil loss rate, land cover change (cropland and bare land) and the area's soil type may cause sediment production. This result agrees with the outcome of Aneseyee et al. (2020).

## 5 Conclusions

Soil loss is a major problem on the earth that affects agriculture production. Investigating soil loss using a spatial explicit model is vital to understand the situation and target priority areas for management measures. The objective of this study was to simulate the spatiotemporal changes of soil loss and sediment export for the identification of soil loss hotspot areas and conservation priority areas. The mean soil loss rate and sediment export increased over the last 30 years (1991–2021). The mean annual soil loss was 23 ton/ha/yr. This rate is above the soil loss tolerable rate. On average, about 36.8 million ton/year of soil was lost each year from the watershed. About 5.8 million ton/yr of soil accumulates in the water body as sediment in the study catchment.

Based on the FAO standard, 22 % of the study area was grouped in the severity class, including SW-4, SW-5, and SW-13. Those sub-watersheds could be taken as the priority (I) for soil and water conservation. The two sub-watersheds such as SW-1 and SW-6 would be the priority (II); it covers 62 % of the study area. The rest of the sub-watersheds would take priority (III), covering 16 % of the study area. Therefore, it is better to prioritize severely affected sub-watersheds by soil loss for SWC measures that will help to restore and sustain the functionality of the catchment.

## Declarations

### Author contribution statement

Chakoro Tamire: Conceived and designed the experiments; Performed the experiments; Analyzed and interpreted the data; Contributed reagents, materials, analysis tools or data; Wrote the paper.

Eyasu Elias: Conceived and designed the experiments; Performed the experiments; Contributed reagents, materials, analysis tools or data; Wrote the paper.

Mekuria Argaw: Performed the experiments; Contributed reagents, materials, analysis tools or data; Wrote the paper.

### Funding statement

This research did not receive any specific grant from funding agencies in the public, commercial, or not-for-profit sectors.

### Data availability statement

Data included in article/supp. material/referenced in article.

### Declaration of interest’s statement

The authors declare no conflict of interest.

### Additional information

No additional information is available for this paper.
